# Simulated rich club lesioning in brain networks: a scaffold for communication and integration?

**DOI:** 10.3389/fnhum.2014.00647

**Published:** 2014-08-21

**Authors:** Marcel A. de Reus, Martijn P. van den Heuvel

**Affiliations:** Department of Psychiatry, Brain Center Rudolf Magnus, University Medical Center UtrechtUtrecht, Netherlands

**Keywords:** connectome, brain network, graph theory, diffusion tractography, rich club, lesioning

Brain function depends on effective neural communication and integration across different domains. This exchange of information is facilitated by the “connectome”: the complex network of all neural elements and neural connections of an organism that provides the anatomical foundation for emerging functional dynamics. How the complex wiring of the connectome relates to the demands and constraints placed upon the brain is an important question in neuroscience, receiving attention from a rapidly increasing number of researchers.

One potential aspect of macroscale connectome architecture related to global communication and integration is the existence of “neural hubs,” referring to brain regions that display many connections and thus exhibit a topologically central position in the overall network. In addition to being individually rich in connectivity, hubs in neural systems tend to be also densely interconnected, together forming a central “rich club” (van den Heuvel and Sporns, 2011). Over the past years, several reports on a number of different species (including human, macaque, cat and nematode) have consistently suggested that brain hubs and their rich club connections play an important role in enabling efficient neural communication and integration, constituting a central communication backbone that boosts the functional repertoire of the system (Zamora-López et al., 2010; Crossley et al., 2013; Towlson et al., 2013; de Reus and van den Heuvel, 2013a; van den Heuvel and Sporns, 2013a; Grayson et al., 2014; Mišić et al., 2014; Senden et al., 2014). Since brain hubs have been shown to be implicated in both neurological (Stam et al., 2007; Buckner et al., 2009) and psychiatric (Collin et al., 2013; van den Heuvel et al., 2013) diseases, it is the general hope that a better fundamental understanding of their role in connectome organization may eventually provide insight in the pathology and effects of brain disorders.

In an interesting article recently published in this journal, Andrei Irimia and John Van Horn aimed to further elucidate healthy brain network architecture by pinpointing those neural connections that are critical for the overall organization of the human connectome (Irimia and Van Horn, 2014). Simulating the effects of white matter lesions by removing individual connections from the connectome, the authors report on a scaffold of white matter connections whose disruption is suggested to have significant global-level effects on the brain. However, quite contrary to our expectations, the authors note that “connections between rich club nodes in the human brain overlap only very moderately—and even then, perhaps accidentally—with the core scaffold.”

Here we discuss the apparent incongruity between Irimia and Van Horn's lesioning scaffold and the growing amount of studies suggesting that neural hubs and their connections may form a fundamental architecture for shaping global neural processes. Analyzing new data, we show that the importance of connections assessed by simulated lesioning largely depends on the measures chosen to evaluate the outcome. We further demonstrate that lesioning connections between rich club regions has pronounced effects on two specific measures of communication and integration, both in the human and animal brain.

## Lesioning connections

We concur with Irimia and Van Horn that while much research is geared toward understanding the network features of brain regions (i.e., the *nodes* of the connectome), network properties of the *edges* between brain regions -representing white matter connections- may contain important additional information (van den Heuvel and Sporns, 2013b). The approach of Irimia and Van Horn to quantify how lesioning an individual connection affects the global network structure is an elegant way to shift focus from nodes to edges and provides insight in the role or importance of the disrupted connection (de Reus et al., in press).

In their paper, individual white matter edges were removed, one at a time, across reconstructed brain networks of 110 individuals. Brain networks were derived using diffusion tractography and comprised 165 regions. The effect of the removal of each edge was quantified by comparing four graph measures, being assortativity, characteristic path length, density and transitivity (see Rubinov and Sporns (2010) for an overview), before and after removal of the edge. Next, the observed differences with respect to these four metrics were combined into a single test statistic for each connection, expressing the significance of the effects caused by its removal. After evaluating the effects across all edges of the connectome, the authors found a diverse scaffold of white matter connections with a significant combined effect on the examined global network metrics, presenting (somewhat surprisingly) only little overlap with hub-to-hub rich club connections.

## Choosing metrics

An essential ingredient of the proposed simulated lesioning approach are the network metrics chosen to assess the consequences of the simulated lesions. Unfortunately, it is generally difficult to judge which network metrics reflect “key” features of the brain and are suitable to capture lesion effects. For instance, since it is not always beneficial for a network to be assortative (Zhou et al., 2012), it is unclear whether changes in assortativity (e.g., due to lesions) are relevant for the functioning of brain networks.

**Figure 1:**
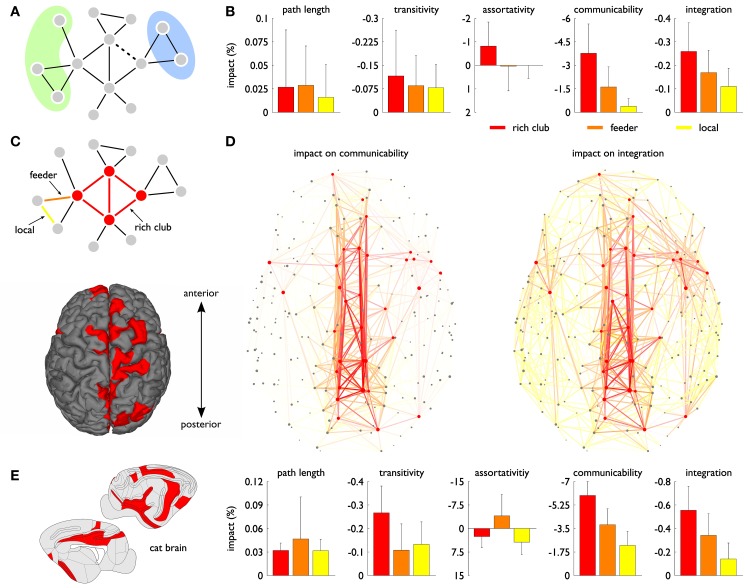
**Effects of simulated connection lesioning**. **(A)** A simple toy network shows that, due to the presence of parallel pathways, removal of the dashed connection does not alter the path length (i.e., minimum number of steps) between nodes in the green and blue zone. A change in communication capacity between the two zones is only detected if also the *number* of possible routes is taken into account, as is the case with Estrada and Hatano's communicability metric (Estrada and Hatano, 2008). **(B)** Impact of connection lesioning in the human brain as measured by five different network metrics. Connection scores were obtained by comparing a group-averaged connectome map, based on high-quality data of 215 subjects from the Human Connectome Project (van Essen et al., 2012), before and after removing an individual edge. The bars and whiskers indicate the average score and standard deviation for the three connection classes illustrated in panel **(C)**: “rich club” connections between (red) rich club nodes, “feeder” connections between rich club and (gray) non-rich club nodes and “local” connections between non-rich club nodes. **(D)** Network plots showing the importance of individual connections for the network's communicability and functional integration. Removal scores are reflected by the transparency of connections, the most important connections being the most opaque. Underscoring the visual concentration of crucial edges around rich club nodes, the vast majority of rich club connections (86%) was found to belong to the 30% connections with the highest impact on communicability. **(E)** Validation on the basis of a tract-tracing reconstruction of the cat connectome revealed highly similar results, again showing a pronounced role of rich club connections in global communication and functional integration.

In addition, there is a somewhat more attestable issue regarding the effectiveness of the mean shortest (i.e., characteristic) path length to assess changes upon connection lesioning. If the hypothesized disruption of a connection does eliminate some but not all shortest paths between two brain regions, the shortest path length between those regions does not change, while their mutual communication is still likely to be affected (Figure 1A). This issue is related to the observation by Rubinov and Sporns (2010) that “measures such as the characteristic path length… do not incorporate multiple and longer paths” and is especially relevant because of the high incidence of parallel processing paths in the brain (Goldman-Rakic, 1988; Alexander and Crutcher, 1990). A generalization of shortest path length that takes *all* possible paths between brain regions into account is the “communicability” metric of Estrada and Hatano (2008). This metric assigns higher weights to shorter paths (see the Supplementary Material for an explicit definition), which elegantly aligns with a recent report showing that shorter communication paths are associated with higher functional connectivity between brain regions (Goñi et al., 2014). Combined with some interesting applications of communicability in neuroscience (Duarte-Carvajalino et al., 2012; Mantzaris et al., 2013), especially as a tool to measure effects of actual (non-simulated) lesions in the brain (Crofts and Higham, 2009; Crofts et al., 2011), we believe that communicability may be a promising metric for simulated lesioning approaches.

## Role of rich club connections

To offer quantitative insight on this matter, we made a connectome map of the human cerebral cortex on the basis of high-quality diffusion-weighted MRI data from 215 subjects as provided by the Q3 data release of the Human Connectome Project (van Essen et al., 2012; Glasser et al., 2013). White matter fibers were traced using generalized q-sampling imaging (GQI; allowing for the reconstruction of crossing fibers) and streamline tractography (Yeh et al., 2010) and the cortex was parcellated into 219 distinct regions on the basis of a high-resolution subdivision of FreeSurfer's Desikan-Killiany atlas (Cammoun et al., 2012). Combining data from all 215 subjects, a group-averaged connectome map was formed by placing an edge between two brain regions if those regions were found to be connected in at least 60% of the subjects (de Reus and van den Heuvel, 2013b). Rich club regions were taken to include the 15% highest degree nodes of this connectome map (van den Heuvel et al., 2012).

### Impact on assortativity, transitivity and path length

As shown in Figure 1B, our analyses revealed that systematic removal of connections between rich club regions did have a (modestly) larger impact on the assortativity and transitivity of the network than removal of “feeder” and “local” connections -respectively reflecting edges between rich club hub regions and peripheral non-hub regions and edges between peripheral regions (Figure 1C). However, in agreement with the report of Irimia and Van Horn, removal of rich club connections did not have an outspoken effect on the characteristic path length of the network (Figure 1B), explaining why rich club connections may not appear in a scaffold that is based on the combined effects on assortativity, transitivity and path length.

### Impact on communicability

Interestingly, the same lesioning approach did reveal a strong impact of rich club connections on the above described communicability measure. In fact, removal of rich club connections (mean [std]: −3.8 [1.9]%) had a 2.4 times larger impact on the network's communicability than removal of feeder connections (mean [std]: −1.6 [1.3]%, *p* < 10^−4^, permutation test) and a 9.5 times larger impact than removal of local connections (mean [std]: −0.4 [0.5]%, *p* < 10^−4^) (Figure 1B).

Furthermore, going beyond the examination of rich club connections as a class, Figure 1D shows the communicability impact for each connection separately, confirming a strong concentration of high-impact edges around rich club nodes. Quantifying the overlap between rich club connections and potential communicability scaffolds, we found that the vast majority of rich club connections (86%) belonged to the 30% connections with the highest impact on communicability, with 79/63% of the rich club connections even scoring among the “best” 20/10%. Statistical evaluation through random reassignment of connection scores showed that these effects are highly unlikely to occur if the two phenomena are unrelated (all *p* < 10^−6^), in which case the expected number of rich club connections among the 30, 20 and 10% connections with the highest impact on communicability would just be equal to, respectively, 30, 20 and 10%.

### Impact on functional integration

Assessment of the effect of simulated connection disruption on a metric for the integration between 11 previously identified resting-state functional brain networks proposed by Tononi et al. (1994) (see the Supplementary Material for details) revealed a similar pattern. As shown in Figures 1B,D, removal of rich club connections (mean [std]: −0.26 [0.12]%) resulted in a 1.5 times larger decrease of functional integration than removal of feeder connections (mean [std]: −0.17 [0.09]%, *p* < 10^−4^) and a 2.4 times larger decrease than removal of local connections (mean [std]: −0.11 [0.08]%, *p* < 10^−4^).

### Validation

As described in more detail in the Supplementary Material, the elevated impact of rich club connections on the adopted communicability and integration metric was both confirmed using alternative group-averaged connectomes (constructed with “group thresholds” of 30, 45, 75 and 90%) and by simulated lesioning of individual connectome reconstructions (see Supplementary Figures 1 and 2). To further validate our findings, we additionally examined a tract-tracing based reconstruction of the cat connectome. Using the same cat rich club regions and functional domains as described in de Reus and van den Heuvel (2013a), removal of rich club connections showed a distinct impact on the communicability of the cat connectome (mean [std]: −6.0 [1.0]%), significantly exceeding the removal effects of both feeder and local connections (both *p* < 10^−4^, Figure 1E). Closely matching our findings on the human connectome, 62% of the rich club connections scored among the top 10% connections with the highest removal effect on communicability and almost all rich club connections (97%) belonged to the top 30%. Moreover, also the staircase relation for functional integration was clearly reproducible (all differences significant with *p* < 10^−4^).

## Discussion

The observed differences between simulated lesioning results assessed with characteristic path length, which is a conventional measure for communication and integration (Rubinov and Sporns, 2010), and simulated lesioning results assessed with Estrada's communicability or Tononi's integration metric, demonstrate that the answer to the question “which white matter connections cause large global-level effects when hypothetically lesioned?” largely depends on the metrics chosen to evaluate those effects, even if the metrics belong to the same “family.” This is further underscored by a *post-hoc* analysis in which we computed the impact of connection disruption on a measure for diffusion-based communication, estimating the number of steps needed to send a “signal” from one brain region to another under the assumption that the signal moves randomly along the connections of the network (see the Supplementary Material for details). Simulated lesioning outcomes with respect to this diffusion-based communication metric qualitatively differed from both characteristic path length and communicability outcomes, showing a “reverse” staircase with a low impact for rich club connections and a prominent role for local connections (Supplementary Figure 3).

Taken together, our results extend the observations of Irimia and Van Horn by showing that although hub-to-hub rich club connections may not appear in an aggregated lesioning scaffold derived using characteristic path length as communication metric, they do have a high impact on two other specific measures for communication and integration, namely Estrada's communicability and Tononi's integration metric. Rich club connections may thus not be critical for establishing (unique) short paths between remote brain regions, but do appear to play an important role in providing diverse communication paths across the network and integration of information between different functional domains. These observations nicely align with a growing number of reports suggesting that neural hubs and their connections play a central role in neural networks (van den Heuvel and Sporns, 2013b) and recent simulation studies suggesting that the presence of neural rich clubs enhances functional diversity (Senden et al., 2014).

The idea behind communicability that also parallel and longer paths may contribute to exchange of information between brain regions holds the middle between characteristic path length, assuming that signals only follow shortest paths, and diffusion-based metrics, assuming randomly moving signals [which actually tend to get “trapped” in densely connected zones such as the rich club (Rosvall and Bergstrom, 2008)]. In our opinion, this could be a plausible regime for the brain, especially because brain networks do not appear to be specifically optimized for either shortest path-based or diffusion-based communication (Goñi et al., 2013). An important future challenge in the field of connectomics will be to examine the biological relevance of such communication principles and other network measures, making it possible to determine which metrics encode “key” features of the brain's wiring architecture.

### Conflict of interest statement

The authors declare that the research was conducted in the absence of any commercial or financial relationships that could be construed as a potential conflict of interest.
