# Supplementary material for: Simulated rich club lesioning in brain networks: a scaffold for communication and integration?
Source: Front Hum Neurosci. 2014 Aug 21;8:647. doi: 10.3389/fnhum.2014.00647 (PMC4139657; doi:10.3389/fnhum.2014.00647)
Supplement: Supplementary file 1 [file DataSheet1.PDF]

Supplementary Material for the article

Simulated rich club lesioning in brain networks: a scaffold for communication and integration?

Marcel A. de Reus and Martijn P. van den Heuvel

Brain Center Rudolf Magnus, Department of Psychiatry, University Medical Center Utrecht, 3584 CX Utrecht, The Netherlands

Communicability and integration

Complementing standard network metrics such as characteristic path length, transitivity and assortativity (see Rubinov and Sporns (2010) for an overview), we also used the following less well-known metrics to quantify the effects of simulated white matter lesions:

Communicability

Originally introduced by Estrada and Hatano (2008), *communicability* G_{ij} is a measure for the 'ease of communication' between two nodes i and j of a network. Rather than taking only the shortest paths between i and j into account, G_{ij} incorporates all possible *walks* (i.e., paths that may visit the same edge multiple times) between the two nodes with appropriate weights, assigning lower weights to longer walks. It is formally given by:

$$G_{ij}=\sum_{k=0}^{\infty}\frac{(A^k)_{ij}}{k!}=(e^A)_{ij},$$

where A denotes the adjacency matrix of the network, satisfying $A_{ij} = 1$ if node i and j are connected and $A_{ij} = 0$ otherwise, and $(A^k)_{ij}$ equals the number of walks of length k from node i to node j. To measure the overall communicability G within the entire network, G_{ij} was averaged over all node pairs (i, j) (Benzi and Klymko, 2013). As such, G is a direct generalization of the characteristic path length, with the notable difference that also the number of shortest paths (i.e., parallel paths), as well as slightly longer paths, are assumed to contribute to information exchange between network nodes.

Integration

As proposed in the seminal paper of Tononi et al. (1994), the level of integration I between subnetworks $X_1, X_2, \ldots, X_m$ of a network X can be theoretically described by the formula:

$$I(X) = \sum_{i=1}^{m} H(X_i) - H(X),$$

where $H(X_i)$ is the entropy of subnetwork X_i, considered as an isolated system, and $H(X)$ is the joint entropy of the network as a whole. The idea behind this formula is that dependencies between the subnetworks reduce the 'amount of freedom' in the network, causing the entropy of the entire system to be lower than the sum of isolated subnetwork entropies. By measuring the size of this discrepancy, $I(X)$ thus effectively measures the extent to which the subnetworks $X_1, X_2, \ldots, X_m$ depend on each other.

As described in detail by Zamora-López et al. (2010), the entropies required to compute $I(X)$ can be derived from the covariance matrix COV(X) of the network. Using a simple linear model for network dynamics in which the nodes of the network are driven by Gaussian noise, this covariance matrix can be computed analytically from the adjacency matrix A and coupling factor g by COV(X) = $Q \cdot Q^t$, with matrix Q equal to $1/(1 - gA^t)$. The entropies $H(X)$, $H(X_1), H(X_2), \ldots, H(X_m)$, and thus I(X), can then be computed using:

$$H(X_i) = \frac{1}{2} \log\left((2\pi e)^{n_i} \left|\mathrm{COV}(X_i)\right|\right),$$

where n_i is the number of nodes in X_i and $|\mathrm{COV}(X_i)|$ is the determinant of the submatrix of COV(X) obtained by selecting the rows and columns that correspond to nodes in X_i.

For the present study, the subnetworks $X_1, X_2, \ldots, X_m$ were taken to reflect 11 previously identified resting-state functional brain networks (van den Heuvel et al., 2014; van den Heuvel and Sporns, 2013) and the coupling factor g was taken to be $0.5/\lambda_{max}$, with λ_{max} equal to the largest eigenvalue of the intact adjacency matrix (Zamora-López et al., 2010).

Mean first-passage time

Next to characteristic path length and communicability, a different perspective on communication in complex networks is given by 'diffusion-based' communication metrics such as the mean first-passage time (Goñi et al., 2013). The assumption behind these diffusion-based metrics is that network nodes do not have any information about each other's (topological) position and possible communication routes, implying that 'signals' have to reach their destination by moving randomly along the connections of the network. The mean first-passage time T_{ij} estimates how many connections a randomly moving signal starting at node i on average has to traverse to reach node j.

Mathematically, random signal movement is captured by the concept of a 'random walk', moving from one node in the network to the next by randomly selecting one of the node's neighbors. The probability that a random walk transits from node i to node j is therefore given by $P_{ij} = A_{ij}/d_i$, with A equal to the adjacency matrix of the network and d_i equal to the degree (i.e., number of connections) of node i. Together, these probabilities P_{ij} constitute the transition matrix P of the network and the mean first-passage time T_{ij} can be computed from P using:

$$T_{ij} = \frac{Z_{jj} - Z_{ij}}{\pi_j},$$

where π is the left eigenvector of P corresponding to its largest eigenvalue (normalized such that $\sum_i \pi_i = 1$), describing the ergodic node visit frequencies of an infinitely long random walk, and $Z = (I - P + W)^{-1}$ is the fundamental matrix, with I equal to the identity matrix and W a square matrix with every row equal to π (Goñi et al., 2013; Zhang et al., 2011). Analogous to the approach for path length and communicability, a global estimate T for the mean first-passage time was obtained by averaging T_{ij} over all node pairs (i, j).

Group threshold selection

Simulated lesioning results were obtained by removing individual connections from a group-averaged connectome map, incorporating high-quality MRI data of 215 subjects as provided by the Human Connectome Project. The group-averaged connectome map was formed by placing an edge between two brain regions if those regions were found to be connected in at least 60% of the subjects. An extensive study concerning this averaging procedure and the associated choice of 'group threshold' was recently published as a separate article (de Reus and van den Heuvel, 2013). In that article, we conclude that the group threshold should preferable be chosen between 30% and 90% and that 60% appears to be an 'optimal' choice, explaining our current setting. To eliminate the possibility that the here presented findings depend on the chosen group threshold, we additionally computed the impact of connection lesioning for group thresholds of 30%, 45%, 75% and 90%. The results obtained using these four additional group thresholds (see Supplementary Figure 1) were found to be highly similar to the results presented in the main text, consistently showing that removal of rich club connections has a significantly larger impact on the above described communicability and integration metric than removal of feeder and local connections.

Simulated lesioning in individual subjects

In addition to simulating lesions in group-averaged connectomes, we also quantified the effects of simulated lesioning on the basis of individual connectome reconstructions. To this end, the average impact of rich club, feeder and local connections with respect to the adopted network metrics was computed for each individual subject and the three resulting distributions were tested for differences using permutation tests (Supplementary Figure 2). Consistent with our findings based on group-averaged connectomes, simulated lesioning of rich club connections had significantly larger impact on communicability and integration than removal of feeder and local connections ($p < 10^{-4}$).

References

Benzi, M. and Klymko, C. (2013). Total communicability as a centrality measure. *J. Complex Networks* 1, 124-149.

Estrada, E. and Hatano, N. (2008). Communicability in complex networks. *Phys. Rev. E* 77, 036111.

Goñi, J., Avena-Koenigsberger, A., Velez de Mendizabal, N., van den Heuvel, M.P., Betzel, R.F. and Sporns, O. (2013). Exploring the Morphospace of Communication Efficiency in Complex Networks. *PLoS ONE* 8, e58070.

van den Heuvel, M.P., Kersbergen, K.J., de Reus, M.A., Keunen, K., Kahn, R.S., Groenendaal, F., de Vries, L.S. and Benders, M.J. (2014). The Neonatal Connectome During Preterm Brain Development. *Cereb. cortex*, doi: 10.1093/cercor/bhu095.

van den Heuvel, M.P. and Sporns, O. (2013). An Anatomical Substrate for Integration among Functional Networks in Human Cortex. *J. Neurosci.* 33, 14489-14500.

de Reus, M.A. and van den Heuvel, M.P. (2013). Estimating false positives and negatives in brain networks. *NeuroImage* 70, 402-409.

Rubinov, M. and Sporns, O. (2010). Complex network measures of brain connectivity: Uses and interpretations. *NeuroImage* 52, 1059-1069.

Tononi, G., Sporns, O. and Edelman, G.M. (1994). A measure for brain complexity: relating functional segregation and integration in the nervous system. *Proc. Natl. Acad. Sci.* 91, 5033-5037.

Zamora-López, G., Zhou, C. and Kurths, J. (2010). Cortical hubs form a module for multisensory integration on top of the hierarchy of cortical networks. *Front. Neuroinform.* 4, 1.

Zhang, Z., Julaiti, A., Hou, B., Zhang, H. and Chen, G. (2011). Mean first-passage time for random walks on undirected networks. *EPJ B* 84, 691-697.

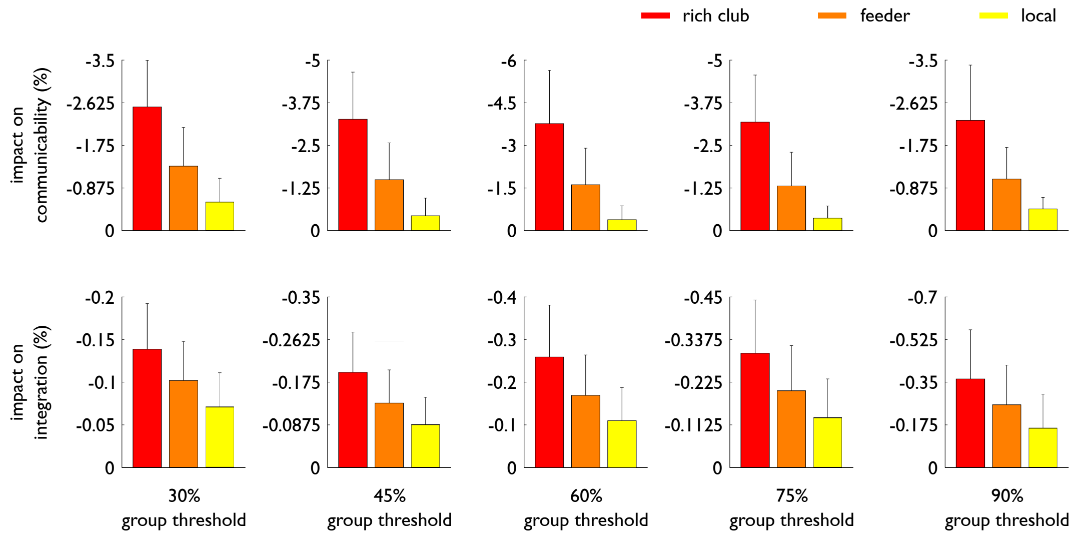

Supplementary Figure 1. Effects of simulated connection lesioning on communicability and integation for five different group-averaged connectomes, constructed with group thresholds of 30%, 45%, 60% (the setting used in the main text), 75% and 90%. For all thresholds, simulated lesioning of rich club connections had a significantly larger impact on communicability and integration than simulated lesioning of feeder or local connections ($p < 10^{-4}$).

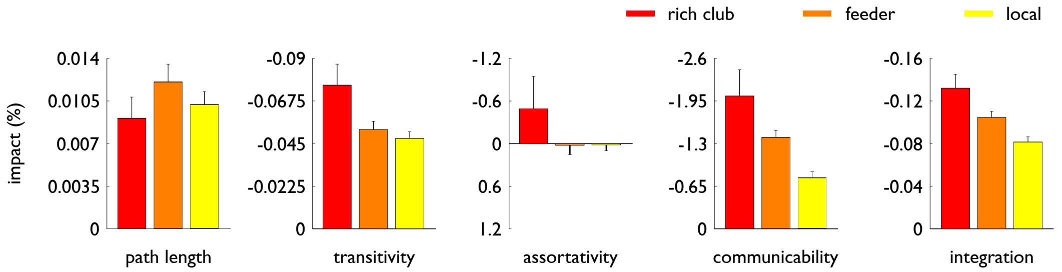

Supplementary Figure 2. Effects of simulated connection lesioning based on individual connectome reconstructions. For each of the 215 subjects, the average impact of its rich club, feeder and local connections was measured with respect to five different network metrics, resulting in 15 distributions of 215 values. The depicted bars and whiskers respectively reflect the mean and standard deviation of these distributions.

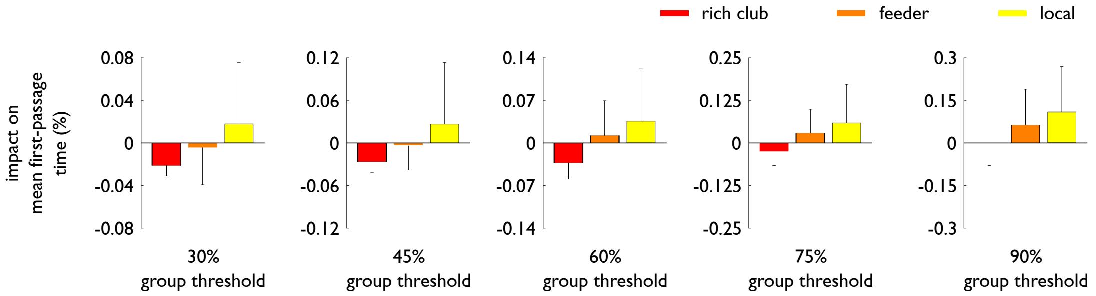

Supplementary Figure 3. Effects of simulated connection lesioning on the mean first-passage time. The mean first-passage time between two nodes *i* and *j* of the network is an estimate for the number of connections that a signal starting at node *i* traverses before it reaches node *j* when randomly moving along the connections of the network.
